# Digital model of biochemical reactions in lactic acid bacterial fermentation of simple glucose and biowaste substrates

**DOI:** 10.1016/j.heliyon.2024.e38791

**Published:** 2024-10-01

**Authors:** Arman Arefi, Barbara Sturm, Majharulislam Babor, Michael Horf, Thomas Hoffmann, Marina Höhne, Kathleen Friedrich, Linda Schroedter, Joachim Venus, Agata Olszewska-Widdrat

**Affiliations:** a Department of Systems Process Engineering, Leibniz Institute for Agricultural Engineering and Bioeconomy (ATB), Max-Eyth Allee 100, 14469, Potsdam, Germany; b Leibniz Institute for Agricultural Engineering and Bioeconomy (ATB), Max-Eyth Allee 100, 14469, Potsdam, Germany; c Albrecht Daniel Thaer-Institute of Agricultural and Horticultural Sciences, Humboldt University of Berlin, Hinter der Reinhardtstr, Germany; d Department of Data Science in Bioeconomy, Leibniz Institute for Agricultural Engineering and Bioeconomy (ATB), Max-Eyth Allee 100, 14469, Potsdam, Germany; e Department of Agromechatronics, Leibniz Institute for Agricultural Engineering and Bioeconomy (ATB), Max-Eyth Allee 100, 14469, Potsdam, Germany; f University of Potsdam, Potsdam, Germany; g Department of Microbiome Biotechnology, Leibniz Institute for Agricultural Engineering and Bioeconomy (ATB), Max-Eyth Allee 100, 14469, Potsdam, Germany

**Keywords:** Near and mid infrared spectroscopy, Lactic acid fermentation, Biowaste fermentation, Deep learning, Non-invasive measurements

## Abstract

As concerns about the environmental impacts of biowaste disposal increase, lactic acid bacterial fermentation is becoming increasingly popular. Current academic research is aimed at the process optimization by developing digital bioreactors. The primary focus is to develop a digital model mimicking the biochemical reactions. In the light of this, this paper intended to build a digital model of biochemical reactions during the fermentation process of both glucose and biowaste substrates, including white pasta and organic municipal waste. For this purpose, near-infrared (NIR) and mid-infrared (MIR) spectroscopy techniques were used to collect spectral information during the fermentation process. Next, the samples were analyzed by High Pressure Liquid Chromatography (HPLC) to measure their glucose, fructose, arabinose, xylose, disaccharide, lactic acid, and acetic acid contents. The results showed that learning algorithms trained on MIR spectra accurately estimated the biochemical reactions for both glucose and biowaste substrates. For the glucose substrate, the results showed R-squared of 0.97 and RMSE of 4.69 g/L for glucose, and R-squared of 0.98 and RMSE of 2.74 g/L for lactic acid. In the case of biowaste substrate, estimations included glucose (R-squared = 0.97, RMSE = 4.69 g/L), fructose (R-squared = 0.88, RMSE = 1.47 g/L), arabinose (R-squared = 0.98, RMSE = 0.55 g/L), xylose (R-squared = 0.93, RMSE = 1.11 g/L), disaccharide (R-squared = 0.90, RMSE = 0.55 g/L), total sugar (R-squared = 0.98, RMSE = 3.79 g/L), lactic acid (R-squared = 0.98, RMSE = 2.74 g/L), and acetic acid (R-squared = 0.97, RMSE = 0.36 g/L). Regarding NIR spectral data, the predictive models were accurate when the substrate was glucose, however, they failed to accurately estimate the chemical reactions in the case of biowaste substrate. The findings of this study can be used to fulfill the requirements for a continuous fermentation process with the objective of maximizing lactic acid production.

## Highlights

• NIR and MIR sceptroscopies were used to monitor fermentation process of biowastes.
• MIR responded to the biochemical reactions accuratly.
• NIR faild to deleiver relaibale information on biochemical reactions.
• Sugars and acids were accuratly predicted by MIR-based machine learning models.

## Graphical abstract

**Figure:**
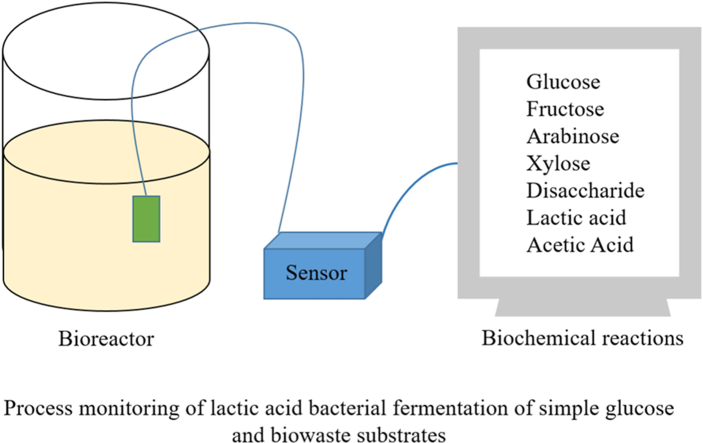

## 1 Introduction

Lactic acid, as a biochemical molecule, is of high interest in different industrial and biomedical applications ranging from polylactic acid production, food, and beverages to pharmaceuticals. Its application is gaining even more attention as it can be polymerized to produce green, biodegradable, and biocompatible packing material, fibers, and foams [1]. Lactic acid is produced either chemically or through fermentation routes. The former is based on petrochemical resources raising a flag due to the environmental concerns, whilst the microbial fermentation is associated with lower energy intake, inexpensive substrates, and environmental friendliness. Furthermore, the microbial pathway can produce either D- or L-lactic acid isomers as opposed to the chemical route producing a mixture of racemic DL-lactic acid [1]. Lactic acid can be fermented from either simple sugars e.g. glucose and sucrose, or organic production side streams and biowaste e.g. lignocellulose, paper slurry, coffee pulp, and kitchen waste [1,2]. Utilization of biowaste substrates is in line with the emerging concept of sustainable circular bio-economy and biorefinery, not only reducing production costs but also addressing waste management issues [3]. To optimize the fermentation process, it is of utmost necessity to monitor biochemical reactions. In this context, near-infrared (NIR) and mid-infrared (MIR) spectroscopies have been of interest. NIR lays between 780 nm and 2500 nm associated with molecular overtones and combinations. Whilst MIR extends to 400 cm^−1^ (25000 nm) related to fundamental vibrations. NIR spectroscopy has been reported as an effective method for monitoring the fermentation process in glucose medium, enabling accurate estimation of lactic acid (r = 0.9988), glucose (r = 0.9971), and biomass (r = 0.9870), [4]. In 1997, the fermentation process monitoring of lactose medium by MIR resulted in standard errors of 3.4, 1.5, 0.9, and 0.9 g/L for lactose, galactose, lactic acid, and bacterial concentration, respectively [5]. [6] monitored the bacterial fermentation of glucose medium by NIR, MIR, and Raman spectroscopies. The MIR-based prediction models outperformed NIR and Raman in the estimation of glucose (R-squared = 0.98 and standard error = 2.77 g/L), lactic acid (R-squared = 0.98 and standard error = 0.53 g/L), and biomass (R-squared = 0.93 and standard error = 0.32 g/L). In another study, the concentration of lactic acid during the fermentation process of glucose-sucrose, glucose-fructose, and glucose-maltose substrates were accurately estimated (R-squared = 0.965 and RMSE = 0.2632 g/L) using MIR spectroscopy [7]. Moreover, NIR spectroscopy was found potential in the quantification of lactic acid (R-squared = 0.989 and RMSE = 3.650) and glucose (R-squared = 0.995 and RMSE = 4.752 g/L) during the fermentation process of glucose medium [8]. Recently, in-situ NIR monitoring of the fermentation process in glucose medium was reported for the estimation of glucose (R-squared = 0.9591 and RPD = 4.9450) and lactic acid (R-squared = 0.9810 and RPD = 7.2525), [9]. NIR (700–1800 nm) was used for the inline fermentation process monitoring; biomass, glucose, lactic acid, and acetic acids were accurately estimated [10]. The process monitoring of yogurt fermentation was successfully carried out by NIR [11] and MIR [12]. Moreover, NIR spectroscopy was reported as a promising tool for real-time monitoring of milk lactic acid fermentation [13]. According to Ref. [14], the peak at 1336 cm⁻^1^ serves as an identification marker of Lactobacillus casei lactic acid bacteria, whilst the spectral region of 1200-900 cm^−1^ indicated the hydrolysis and metabolism during fermentation. In another study, MIR spectroscopy was used to estimate the density (RMSE = 0.0014 g/mL) and pH (RMSE = 0.06 g/mL) parameters in a wine fermentation process [15].

Current state-of-the-art is limited to monitoring fermentation processes using monosaccharides or disaccharides as substrates. The novelty of this study is to approach monitoring biochemical reactions when organic municipal waste and pasta waste hydrolysates were used as substrates. Unlike monosaccharide or disaccharide media, the biowaste hydrolysates present a more complex composition. To tackle this challenge, the study aims to achieve the following specific objectives.

- NIR and MIR spectral measurements of glucose and biowaste hydrolysates during the fermentation process.
- NIR and MIR spectral interpretation.
- Development of prediction models for lactic acid, acetic acid, glucose, xylose, disaccharide, arabinose, fructose, and total sugar.

## 2 Material and methods

### 2.1 Media, culture conditions, and HPLC measurements

All microbial strains used in this study were L-LA producing bacteria that belong to the strain collection of the Leibniz Institute for Agricultural Engineering and Bioeconomy (ATB), Potsdam, Germany. Pre-cultures were cultivated in MRS broth (Merck, Germany) with dolomite EVERZIT Dol 0.5–2.5 mm (Evers, Germany) as a buffering system. The pre-culture preparation and fermentation conditions of pasta and organic municipal waste hydrolysates are described in Refs. [16,17], and [18]. Concentration of glucose, xylose, arabinose, fructose, disaccharides, lactic acid, and acetic acid were measured via High Pressure Liquid Chromatography (HPLC; DIONEX Sunnyvale, CA, USA) as described in Ref. [17]. Besides pasta and organic municipal waste hydrolysates, fermentation process of glucose medium was also carried out as described in Ref. [19]. Table 1 gives an overview of fermentation substrates and biochemical reactions.

**Table 1:** An overview of fermentation substrates and biochemical reactions.

Substrate	Components	Concentration (g/L)			
		Minimum	Maximum	Mean	Standard deviation
Glucose	Glucose	0.00	117.96	45.46	38.12
	Lactic acid	0.00	91.46	40.14	29.76
Pasta waste hydrolysate	Glucose	0.00	143.72	46.88	36.93
	Disaccharide	0.00	4.38	0.84	0.77
	Fructose	0.00	10.35	0.38	1.81
	Xylose	0.00	11.35	0.40	1.99
	Arabinose	0.00	4.05	0.14	0.71
	Total sugar	0.00	145.54	48.49	36.92
	Lactic acid	0.00	68.92	15.16	20.13
	Acetic acid	0.00	1.11	0.05	0.20
Organic municipal waste hydrolysate	Glucose	0.00	57.99	24.46	17.75
	Disaccharide	0.00	8.68	2.32	2.28
	Fructose	0.00	16.00	2.81	4.88
	Xylose	0.00	13.62	1.70	2.33
	Arabinose	0.00	18.27	1.80	4.44
	Total sugar	0.49	71.62	27.39	18.26
	Lactic acid	0.00	77.97	28.76	15.82
	Acetic acid	0.00	60.38	7.46	12.78

### 2.2 NIR and MIR measurements

The samples were first centrifuged to remove solid parts. Next, MIR measurements were carried out using a bench-type FT-IR sensor working in the spectral region of 400–4000 cm^−1^ (Thermo Fisher Scientific, USA). The sensor was first background calibrated, and then, a drop of liquid sample was dripped on the sensor crystal. It was scanned 32 times, and the average was saved as the spectrum. The process was continued by NIR measurements using an FT-NIR sensor working in the spectral range of 775–2600 nm (ARCoptix S.A., Neuchatel, Switzerland). Detailed information about the FT-NIR sensor can be found in Ref. [20]. To collect NIR data, the sample (2.5 ml) was poured into a glass and its spectrum was taken in the reflectance mode. The number of samples used for the NIR and MIR measurements were:

NIR.

− Pasta and organic municipal waste hydrolysates: glucose (608), xylose (592), arabinose (452), fructose (444), disaccharides (513), lactic acid (607), and acetic acid (574).
− Glucose substrate: glucose (579) and lactic acid (580)

MIR.

− Pasta and organic municipal waste hydrolysates: glucose (661), xylose (643), arabinose (496), fructose (471), disaccharides (549), lactic acid (661), and acetic acid (623).
− Glucose substrate: glucose (265) and lactic acid (266)

### 2.3 Spectral processing and learning algorithms

The NIR spectra were corrected using the dark and reference measurements [Eq. (1)]:

(1) $$S_{corr}=\frac{S_{raw}-Dark}{Reference-Dark}\times z$$

where, S_corr_, S_raw_, and z stand for the corrected spectrum, raw spectrum, and the conversion factors for each wavelength from the diffuse reflection standard, respectively. Detailed information about the diffuse reflection standard is given in Ref. [20].

Both NIR and MIR spectra were converted into pseudo absorbance by taking the logarithm of their inverse. The spectra were further processed using the standard normal variate (SNV), first and second derivatives, moving average, multiplicative scatter correction, and Savitzky–Golay filters. In the case of NIR, the spectral range of 800–2450 nm was used in the development of prediction models.

Partial Least Squares Regression (PLSR), Gaussian Process Regression (GPR), Multilayer Perceptron (MLP), and Deep Neural Networks (DNN) were developed in Python with the aim of prediction of lactic acid, acetic acid, glucose, xylose, disaccharide, arabinose, fructose, and total sugar. PLSR is a multivariable regression model extracting latent features from the spectral data supervised by the response biochemical reactions. This method is especially beneficial when dealing with highly collinear predictors or the quantity of predictors surpasses the number of observations. In this paper, PLSR was employed as a baseline model to predict the concentrations of various biochemical substances using raw and preprocessed spectra, where the number of components was selected based on training error. Besides PLSR, Gaussian Process Regression (GPR), a non-parametric method, was applied using a radial basis function kernel. Moreover, a Multilayer Perceptron (MLP) with an input layer, three hidden layers of 20 neurons each, and an output layer was trained with a dropout rate of 0.03. The forth model used in this paper was a Deep Neural Networks (DNN) whose configuration included four hidden layers consisting of 1000, 500, 100, and 10 neurons, and an output layer. In both MLP and DNN, the activation function was Rectified Linear Unit (ReLU) and the learning rate was set to 0.01. For the purpose of training and testing, 75 % and 25 % of the dataset was divided into training and test sets, respectively. The performance of abovementioned models was assessed based on Root Mean Square Error (RMSE) and coefficient of determination (R-squared).

## 3 Results and discussion

### 3.1 Spectral analysis

#### 3.1.1 MIR spectra

MIR spectra are typically divided into four spectral regions: 4000–2500 cm⁻^1^, 2500–2000 cm⁻^1^, 2000–1500 cm⁻^1^, and below 1500 cm⁻^1^, which correspond to X–H stretching, triple bonds, double bonds, and the fingerprint region, respectively [21]. These spectral regions will be used in the following analysis for the purpose of peak assignment.

— Standard chemicals

**Fig. 1:**
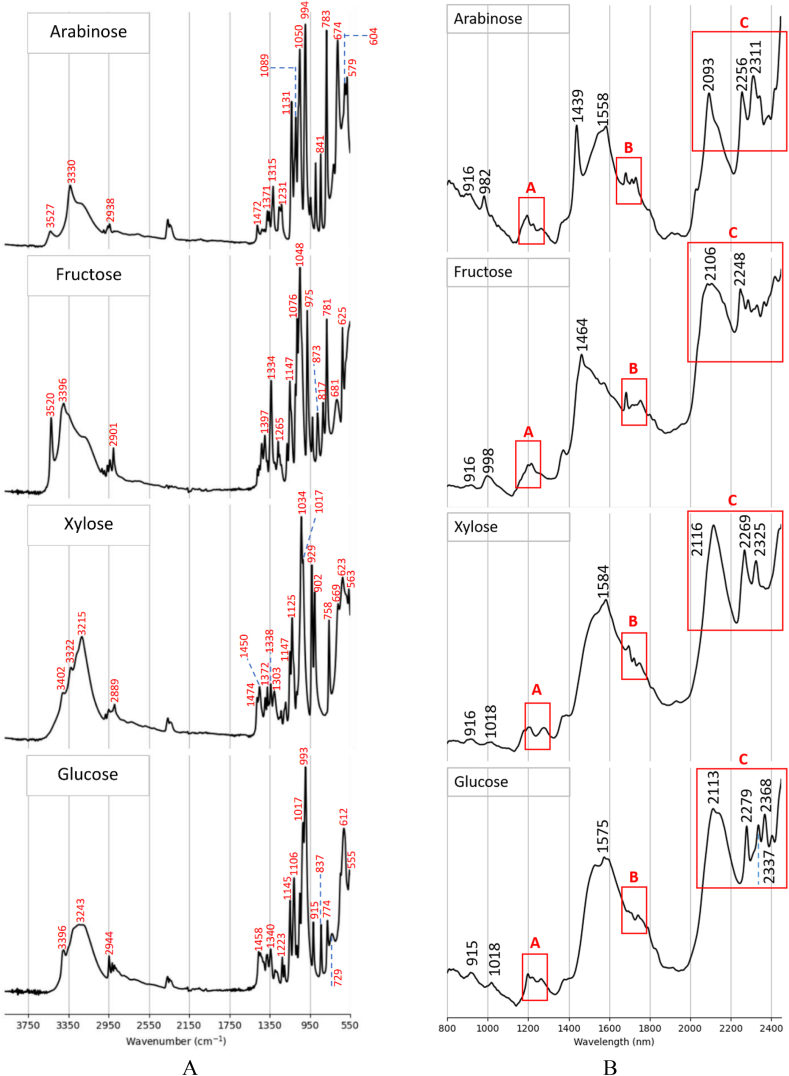
Spectra of standard sugar powders: MIR (A) and NIR (B).

**Fig. 2:**
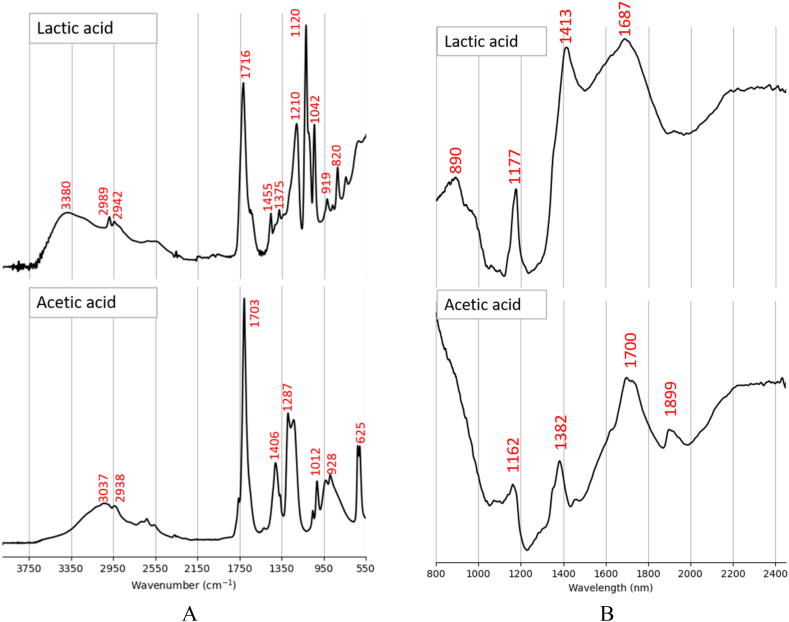
Spectra of standard organic acid liquids: MIR (A) and NIR (B).

To facilitate the spectral interpretation of the fermentation process, MIR spectra of standard chemicals were measured. Fig. 1-A depicts the MIR spectra of standard glucose, fructose, arabinose, and xylose powders. All the sugars showed a broad peak with several shoulders above 3000 cm^−1^, corresponding to the stretching of O–H functional group. The small peaks just below 3000 cm^−1^, i.e. 2944 cm^−1^ for glucose, 2889 cm^−1^ for xylose, 2901 cm^−1^ for fructose, and 2938 cm^−1^ for arabinose, are related to the C–H stretching of the CH_2_/CH groups. The fingerprint region, as expected, contained a high number of peaks. For the glucose, the strongest peak appeared at around 993 cm^−1^ related to the in-plane bending of CCH and CCO groups. The second strongest peak was at 1017 cm^−1^ where C–C stretching, C–O stretching, and COH in-plane bending modes happen. It was followed by a strong peak at 612 cm^−1^ relating to the in-plane bending of CCC, CCO, and OCO groups. Other distinctive peaks in the fingerprint region were 1458 cm^−1^ (CH_2_ scissoring), 1340 cm^−1^ (CH_2_ wagging), 1223 cm^−1^ (CH_2_ twisting), 1145 cm^−1^ (C–O and C–C stretching), 1106 cm^−1^ (C–O and C–C stretching and COH in-plane bending), 915 cm^−1^ (CCH and CCO in-plane bending), 837 cm^−1^ (C–C stretching), and 774, 729, and 555 cm^−1^ (CCC, CCO, and OCO in-plane bending) [22]. Regarding the xylose, the strongest peak in the fingerprint region appeared at 1034 cm^−1^, along with its shoulder at 1017 cm^−1^ related to C–C stretching, C–O stretching, and COH in-plane bending. Two additional predominant peaks were observed at 929 and 902 cm^−1^, associated with the vibrational modes of stretching (COC, C–C, and C–O) and in-plain bending (CCH). The other peaks were related to the stretching mode of C–C and C–O (1147 and 1125 cm^−1^), scissoring, wagging, and twisting modes of CH_2_ (1474–1303 cm^−1^), and in-plane bending of CCC and ring deformation (758–563 cm^−1^). In the case of fructose, the strongest peak appeared at 1048 cm^−1^, corresponding to C–C stretching, C–O stretching, and COH in-plane bending. The peaks at 1397, 1134, and 1147 cm^−1^ were associated with the wagging and twisting modes of CH_2_. The peak at 975 cm^−1^ was related to the in-plane bending of CCH and CCO groups. The peaks at 873 cm^−1^ and 817 cm^−1^ were associated with the stretching mode of C–C. The remaining three peaks at 781, 681, and 625 cm^−1^ were associated with in-plane bending of CCC, CCO, and OCO groups [22]. For the arabinose, four predominate peaks were observed at 1050 cm^−1^ (C–O and C–C stretching, and COH in-plane bending), 994 cm^−1^ (stretching of COC, C–C, C–O and in-plain bending of CCH), 783 and 674 cm^−1^ (C–O stretching and in-plain bending of COC, CCO, and OCO). The peaks between 1472 cm^−1^ and 1231 cm^−1^ were related to the scissoring, wagging, and twisting modes of CH_2_. The peaks at 1131 cm^−1^ and 1089 cm^−1^ corresponded to the C–C and C–O stretching. The peaks below 647 cm^−1^ are associated with the in-plane bending of CCC and ring deformation [22]. Besides sugars, the MIR spectra of standard lactic acid and acetic acid were measured (Fig. 2-A). Lactic acid exhibited a broad peak at 3380 cm⁻^1^ associated with the O–H functional group, followed by two peaks just below 3000 cm⁻^1^ corresponding to C–H stretching [23]. A strong peak appeared at 1716 cm^−1^ corresponding to the C=O stretching mode of carboxylic acid group [7]. The peak at 1454 cm^−1^ corresponded to the asymmetric deformation mode of CH_3_ [23]. A distinctive peak appeared at 1210 cm^−1^, related to the C-O-C asymmetric vibrations [23], was followed by a stronger peak at 1120 cm⁻^1^, which is assigned to CH_3_ rocking [24,25]. The peak at 1375 cm^−1^ corresponded to the symmetric deformation of CH_3_ [25]. The peak at 1042 cm^−1^ is assigned to the vibration of C–CH_3_ [25]. The peak at 820 cm^−1^ was related to the vibration of C–COOH [24]. For the acetic acid, the peaks at 3037 cm^−1^ and 2938 cm^−1^ were related to the stretching of O–H and C–H groups, respectively. The strongest peak, associated with C=O, appeared at 1703 cm^−1^ [26]. It was followed by two distinctive peaks at 1406 cm^−1^ (C–O stretch) and 1287 cm^−1^ (C–O–H in-plane bending), [27]. The peak at 625 cm^−1^ is close to the 637 cm^−1^ peak assigned to OCO bending in acetic acid aerosols [28].

— Glucose fermentation process

**Fig. 3:**
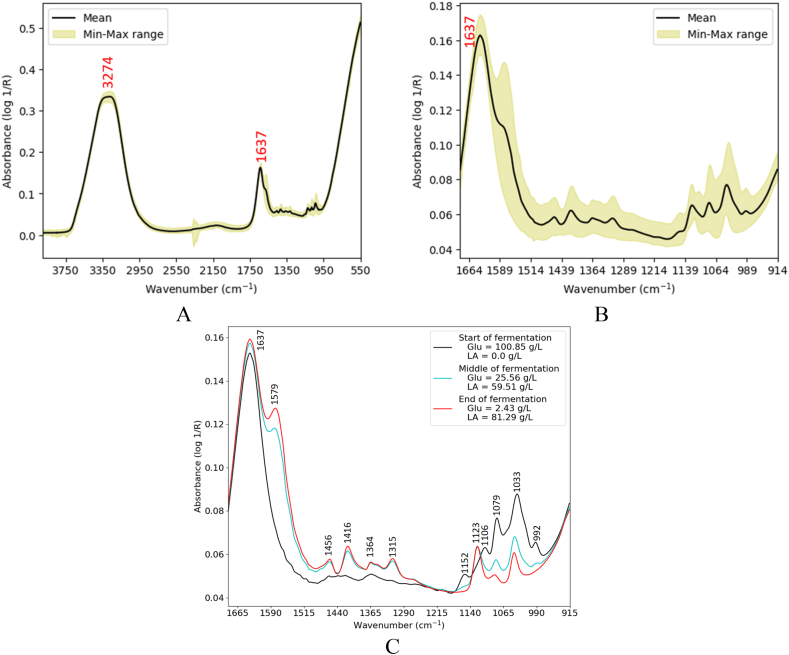
MIR spectra of the glucose fermentation process: full spectrum (A), finger print region (B), spectral changes during the fermentation process (C).

Fig. 3 depicts the MIR spectrum of lactic acid fermentation process with glucose as the substrate. As depicted in Fig. 3-A, two distinctive peaks appeared at 3274 cm^−1^ and 1637 cm^−1^. The former peak is broader due to the overlapping vibrations of O–H groups in water molecules, lactic acid, and glucose. Compared to the MIR spectra of standard sugars and acids shown in Fig. 1-A and Fig. 2-A, the peak is much stronger due to the presence of water molecules. Moreover, the presence of water molecules overshadowed the absorption peaks related to C–H and N–H groups. The latter peak, i.e. 1637 cm^−1^, was most likely related to the amide I band, as reported by Ref. [6]. This is evident from Fig. 3-C, where the peak exhibits high intensity even at the beginning of the fermentation process, before lactic acid was present in the medium. Additionally, the presence of water molecules, which absorb light at 1670 cm⁻^1^ (O–H bending) [29], should also be taken into account. The most interesting and informative spectral region was found in the fingerprint region (Fig. 3-B). A distinctive shoulder appeared at 1579 cm^−1^, most probably associated with the C=O stretching mode of carboxylic acid group [5]. Compared to the corresponding peak in the spectrum of standard lactic acid (Fig. 2-A), it shows a spectral shift of 137 cm⁻^1^. Looking at Fig. 3-C provides greater confidence that the shoulder was associated with lactic acid, as it appeared with the progress of fermentation and not at the start, when lactic acid concentration was absent. The shoulder cannot be attributed to cells, as they were present in the medium from the beginning of the process. The peaks between 1456 cm^−1^ and 1315 cm^−1^ were assigned to the deformation of CH_2_, CH_3,_ and C–OH groups [5]. As shown in Fig. 3-C**,** there were six peaks in the spectral region below 1200 cm^−1^. The peak at 1123 cm⁻^1^ emerged as the fermentation progressed. According to the spectra of standard lactic acid (Fig. 2-A), it can be associated with the CH_3_ rocking mode. The remaining five peaks (1152, 1106, 1079, 1033, and 992 cm⁻^1^) corresponded to changes in glucose concentration during fermentation. Three of the peaks (i.e. 1145, 1106, and 993 cm^−1^) were very close to the peaks in the spectrum of standard glucose (Fig. 1-A).

— Biowaste hydrolysate fermentation process

**Fig. 4:**
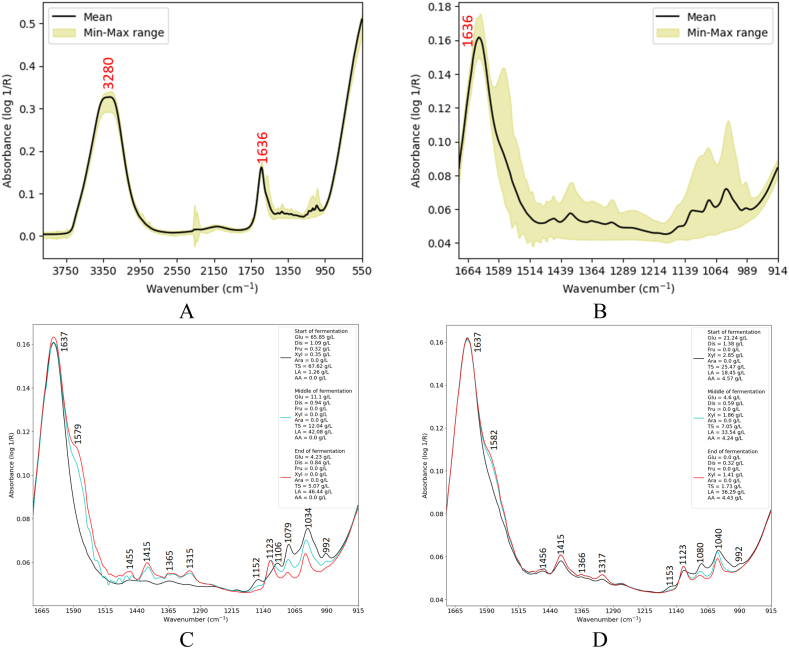
MIR spectra of the biowaste hydrolysate fermentation process: full spectrum (A), finger print region (B), pasta hydrolysate (C), and organic municipal hydrolysate (D).

Fig. 4 depicts the MIR spectra of biowaste hydrolysates. Similar to the MIR spectra of the glucose substrate, two dominant peaks associated with the O–H and amide I groups appeared at 3280 cm⁻^1^ and 1636 cm⁻^1^, respectively (Fig. 4-A). Fig. 4-B depicts the spectral range below 1636 cm^−1^, providing information on sugars and acids. For a clearer understanding, the spectral changes of each biowaste substrate during the fermentation process are shown in Fig. 4-C and Fig. 4-D. Fig. 4-C shows the spectral changes of white pasta hydrolysate. With the fermentation progress of white pasta hydrolysate, the intensity of some peaks (1579, 1455, 1365, 1315, and 1123 cm^−1^) increased, whilst a decrease in the intensity of other peaks (1152, 1106, 1079, 1034, and 992 cm^−1^) was noticed. Such a consistent pattern will facilitate the learning process for algorithms and enhance prediction accuracy, as the peaks provide relevant information on chemical composition. Notably, the similarity between the spectra of white pasta hydrolysate and glucose substrates is interesting. This similarity may be attributed to the high concentration of glucose in the white pasta hydrolysate. The consistent pattern of spectra during the fermentation process was also noticed for the organic municipal waste hydrolysate (Fig. 4-D). However, the spectra differed slightly from those of white pasta; I) the light absorption in the spectral range of 1500–1300 cm⁻^1^ at the beginning of fermentation (black solid line in Fig. 4-D) was higher due to the initial concentration of lactic acid, II) There was no peak observed at 1106 cm⁻^1^, and the peaks at 1153 and 992 cm⁻^1^ were weaker due to the lower initial concentration of glucose.

#### 3.1.2 NIR spectra

— Standard molecules

Fig. 1-B depicts the NIR spectra of standard glucose, fructose, xylose, and arabinose powders. All sugars showed a weak absorption peak at around 916 nm, corresponding to the third overtone of C–H stretching [30]. It was followed by a smaller peak associated with the second overtone of O–H: 1018 nm for glucose and xylose, 998 nm for fructose, and 982 nm for arabinose. The spectral region “A", ca. 1190–1266 nm, showed several small peaks associated with the second overtone of C–H [21]. The peaks at 1439 and 1464 nm were associated with the first overtone of O–H group [31]. A strong peak appeared at 1575 nm for glucose, 1584 nm for xylose, and 1558 nm for arabinose. The peak was assigned to the combination of O–H and C–H stretching by Ref. [32]. The spectral region “B" was related to the first overtone of C–H groups [31]. The strongest absorptivity of sugars occurred in the spectral range “C", above 2100 nm, was associated with O–H, C–H, and C–C combinations [33]. Fig. 2-B shows the NIR spectra of acetic and lactic acid liquids. Acetic acid showed a peak at 1162 nm associated with the second overtone of C–H groups. The next peak appeared at 1382 nm related to hydroxyl group. The strongest peak appeared at 1698 nm, corresponding to the first overtone of C–H groups. A small peak appeared at 1899 nm associated with the combination of C=O and O–H stretching modes [34]. Lactic acid showed a peak at 890 nm, most likely related to the third overtone of C–H. The peaks at 1177, 1413, and 1687 nm were associated with the second overtone of C–H, the first overtone of O–H, and the first overtone of C–H, respectively. As opposed to the standard sugars, no distinctive peak was observed in the spectral range above 2100 nm.

— Glucose fermentation

**Fig. 5:**
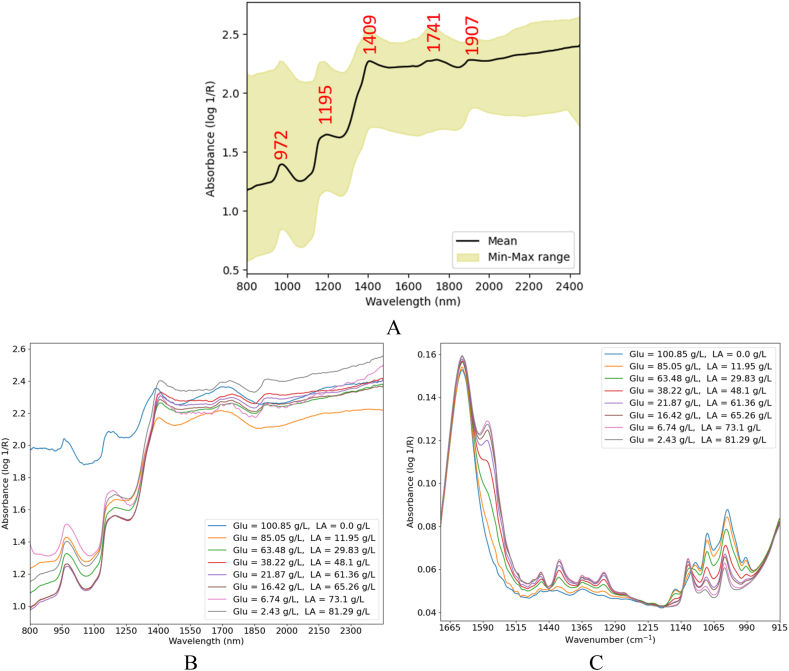
NIR spectra of the glucose fermentation process (A), NIR spectral changes during the fermentation process (B), and MIR spectral changes during the fermentation process (C).

Fig. 5 shows the NIR spectra of fermentation process with the glucose as substrate. As depicted in Fig. 5-A, a peak appeared at 972 nm, corresponding to the O–H groups in glucose, lactic acid, and water. The next distinctive peak appeared at 1195 nm, corresponding to the second overtone of C–H [21], as well as water molecules combination [35]. As expected, a strong peak was observed at 1409 nm associated with the first overtone of O–H bond in glucose, lactic acid, and water. The broad peak at 1741 nm was associated with the first overtone of C–H groups [31]. The last peak appeared at 1907 nm, corresponding to the combination of water molecules [36]. As opposed to the MIR spectra (Fig. 3), it is challenging to assign any specific spectral peak in the NIR region to either lactic acid or glucose, as the glucose molecules also exhibit absorption where lactic acid does. Furthermore, water molecules were active in all the bands associated with lactic acid and glucose molecules, except for the broad band at 1741 nm. Such spectral overlaps not only raised concerns about the certainty of predictions but also complicated the spectral pattern. Fig. 5-B plots the NIR spectral pattern with the progress of fermentation process. Compared to the MIR spectra (Fig. 5-C), where the intensity of characteristic peaks for glucose decreased and lactic acid increased with the progress of fermentation, the NIR spectral changes were mixed. The peak intensities at 972 nm and 1195 nm initially decreased with the progress of fermentation, but later increased. The spectra were primarily influenced by glucose concentration over a longer timeframe compared to lactic acid.

— Biowaste hydrolysate fermentation

**Fig. 6:**
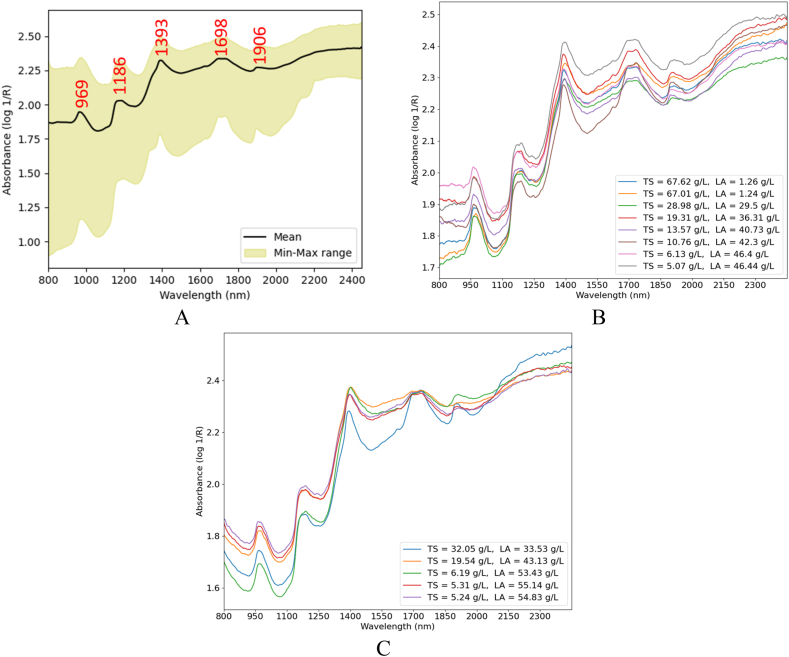
NIR spectra of pasta and organic municipal hydrolysates fermentation process (A), NIR spectral changes during the pasta hydrolysate fermentation process (B), and NIR spectral changes during the organic municipal hydrolysate fermentation process (C).

Fig. 6-A illustrates the NIR spectra of biowaste hydrolysate fermentation. As shown in Fig. 6-B and Fig. 6-C, the spectral patterns for the fermentations of biowaste hydrolysates were mixed. The complexity of the fermentation medium led to random variations in the NIR spectra, complicating further analysis. The mixed pattern may be attributed to the overlapping bands, typical of the NIR region.

### 3.2 Performance of prediction models

#### 3.2.1 MIR-based prediction models

—F020 Glucose fermentation process

Table 2 summarizes the performance of MIR-based learning algorithms in the prediction of biochemical reactions during the fermentation process of glucose substrate. The GPR model achieved the highest prediction accuracy for glucose content, with R-squared values of 1.00 and 0.99, and RMSE values of 0.00 g/L for the training and test datasets, respectively. The prediction accuracies of glucose content achieved by other models were also high, with PLSR showing the lowest accuracy (R-squared = 0.98 and RMSE = 4.77 g/L for the test dataset). MIR-based predictive models were also accurate in the prediction of lactic acid. DNN was the most accurate model (R-squared = 0.99 and RMSE = 2.41 g/L) followed by GPR (R-squared = 0.99 and RMSE = 3.39 g/L).

— Biowaste hydrolysate fermentation process

**Table 2:** Prediction results of chemical components during the glucose fermentation process.

Component	Spectral range	Model	Training		Test	
			R-squared	RMSE (g/L)	R-squared	RMSE (g/L)
Glucose	NIR	**DNN**	**1.00**	**0.00**	**0.84**	**15.34**
		GPR	1.00	0.00	0.78	17.75
		MLP	0.95	8.55	0.81	16.47
		PLSR	0.96	7.66	0.67	21.74
	MIR	DNN	1.00	0.00	0.99	3.43
		**GPR**	**0.99**	**4.55**	**1.00**	**0.00**
		MLP	0.89	12.37	0.99	4.11
		PLSR	1.00	0.82	0.98	4.77
Lactic acid	NIR	**DNN**	**1.00**	**0.00**	**0.93**	**7.87**
		GPR	1.00	0.00	0.91	8.89
		MLP	0.89	9.52	0.88	10.38
		PLSR	0.97	4.95	0.88	10.47
	MIR	**DNN**	**1.00**	**0.00**	**0.99**	**2.41**
		GPR	1.00	0.00	0.99	3.39
		MLP	0.77	14.21	0.98	3.71
		PLSR	1.00	0.22	0.98	4.01

Even though the biowaste hydrolysates brought complexity into the medium, MIR-based learning models were still accurate in the prediction of biochemicals. As presented in Table 3, the glucose content was best predicted by DNN (R-squared = 0.97 and RMSE = 4.69 g/L). The glucose predictions by GPR (R-squared = 0.97 and RMSE = 5.02 g/L), MLP (R-squared = 0.94 and RMSE = 6.70 g/L), and PLSR (R-squared = 0.96 and RMSE = 5.85 g/L) were also accurate. MIR-based DNN model estimated the disaccharide concentration with R-squared and RMSE values of 0.90 and 0.55 g/L, respectively. R-squared (≥0.83) and RMSE (≤0.71 g/L) values achieved by other models were also encouraging. The arabinose content was also accurately estimated; the highest accuracy was achieved by DNN (R-squared = 0.98 and RMSE = 0.55 g/L) followed by MLP (R-squared = 0.97 and RMSE = 0.64 g/L), PLSR (R-squared = 0.96 and RMSE = 0.78 g/L), and GPR (R-squared = 0.95 and RMSE = 0.87 g/L). The highest prediction accuracy of xylose was related to MLP (R-squared = 0.93 and RMSE = 1.11 g/L). Among the sugars, the lowest prediction accuracy was achieved for fructose (0.71 ≤ R-squared ≤0.88 and 1.47 ≤ RMSE ≤2.32 g/L). The prediction models accurately quantified total sugar, with DNN being the most accurate model (R-squared = 0.98 and RMSE = 3.79 g/L). In the case of lactic acid, the highest prediction accuracy was associated with the DNN model (R-squared = 0.98 and RMSE = 2.74 g/L) followed by GPR (R-squared = 0.98 and RMSE = 3.29 g/L). the acetic acid content was best predicted by the GPR model (R-squared = 0.97 and RMSE = 0.36 g/L).

**Table 3:** Prediction results of chemical components during the fermentation process of pasta and organic municipal wastes.

Component	Spectral range	Model	Training		Test	
			R-squared	RMSE (g/L)	R-squared	RMSE (g/L)
Glucose	NIR	**DNN**	**1.00**	**0.00**	**0.21**	**27.83**
		GPR	1.00	0.00	0.07	30.28
		MLP	0.88	10.82	0.13	29.26
		PLSR	0.87	11.24	0.00	34.45
	MIR	**DNN**	**1.00**	**0.00**	**0.97**	**4.69**
		GPR	1.00	0.00	0.97	5.02
		MLP	0.88	10.75	0.94	6.70
		PLSR	0.98	4.83	0.96	5.85
Disaccharide	NIR	**DNN**	**1.00**	**0.00**	**0.45**	**1.26**
		GPR	1.00	0.00	0.42	1.30
		MLP	0.61	1.06	0.55	1.14
		PLSR	1.00	0.08	0.00	1.77
	MIR	**DNN**	**1.00**	**0.00**	**0.90**	**0.55**
		GPR	1.00	0.00	0.85	0.68
		MLP	0.82	0.72	0.83	0.71
		PLSR	0.96	0.35	0.83	0.71
Arabinose	NIR	DNN	1.00	0.00	0.78	1.98
		GPR	1.00	0.00	0.51	2.93
		**MLP**	**0.53**	**2.98**	**0.82**	**1.77**
		PLSR	1.00	0.15	0.47	3.03
	MIR	**DNN**	**1.00**	**0.23**	**0.98**	**0.55**
		GPR	1.00	0.00	0.95	0.87
		MLP	0.97	0.70	0.97	0.64
		PLSR	1.00	0.05	0.96	0.78
Xylose	NIR	DNN	1.00	0.00	0.31	2.67
		GPR	1.00	0.00	0.08	3.09
		**MLP**	**0.87**	**1.47**	**0.38**	**2.53**
		PLSR	1.00	0.18	0.00	3.59
	MIR	DNN	1.00	0.00	0.91	1.26
		GPR	1.00	0.00	0.92	1.17
		**MLP**	**0.95**	**0.84**	**0.93**	**1.11**
		PLSR	0.99	0.38	0.89	1.38
Fructose	NIR	DNN	1.00	0.00	0.31	3.86
		GPR	1.00	0.00	0.38	3.67
		**MLP**	**0.47**	**3.42**	**0.48**	**3.37**
		PLSR	1.00	0.03	0.14	4.31
	MIR	**DNN**	**1.00**	**0.00**	**0.88**	**1.47**
		GPR	1.00	0.00	0.77	2.05
		MLP	0.96	0.96	0.74	2.18
		PLSR	1.00	0.05	0.71	2.32
Total sugar	NIR	DNN	1.00	0.00	0.28	27.42
		GPR	1.00	0.00	0.28	27.40
		**MLP**	**0.64**	**18.37**	**0.33**	**26.40**
		PLSR	0.85	11.97	0.00	32.44
	MIR	**DNN**	**1.00**	**0.00**	**0.98**	**3.79**
		GPR	1.00	0.00	0.97	4.54
		MLP	0.89	10.09	0.94	6.64
		PLSR	0.97	5.11	0.97	4.34
Lactic acid	NIR	**DNN**	**1.00**	**0.00**	**0.35**	**17.95**
		GPR	1.00	0.00	0.22	19.69
		MLP	0.8	8.67	0.25	19.32
		PLSR	0.86	7.19	0.05	21.70
	MIR	**DNN**	**1.00**	**0.00**	**0.98**	**2.74**
		GPR	1.00	0.00	0.98	3.29
		MLP	0.89	6.40	0.97	3.79
		PLSR	0.98	3.03	0.98	3.37
Acetic acid	NIR	**DNN**	**1.00**	**0.00**	**0.54**	**1.50**
		GPR	1.00	0.00	0.44	1.66
		MLP	0.61	1.26	0.51	1.55
		PLSR	1.00	0.04	0.21	1.97
	MIR	DNN	1.00	0.01	0.94	0.53
		**GPR**	**1.00**	**0.00**	**0.97**	**0.36**
		MLP	0.88	0.69	0.95	0.51
		PLSR	0.99	0.15	0.96	0.46

#### 3.2.2 NIR-based prediction models

— Glucose fermentation process

Table 2 summarizes the prediction results for the glucose substrate case. Overall, the prediction accuracies were lower than those associated with MIR. The concentration of glucose content was best predicted by the DNN model (R-squared = 0.84 and RMSE = 15.34 g/L). The Lactic acid content was predicted more accurately than the glucose content. The highest prediction accuracy for lactic acid was achieved by DNN (R-squared = 0.93 and RMSE = 7.87 g/L). PLSR was associated with the lowest prediction accuracy for lactic acid (R-squared = 0.88 and RMSE = 10.47 g/L).

— Biowaste hydrolysate fermentation process

Table 3 presents the performance of NIR-based learning algorithms associated with the biowaste hydrolysate. Overall, the models failed to generate good predictions for the sugars and acids contents. This result was expected given the random pattern observed in the NIR spectra (Fig. 6). The glucose content was best predicted by the DNN model (R-squared = 0.21 and RMSE = 27.83 g/L). The performance of other models was even worse. The prediction of disaccharide content was more accurate than that of glucose but remained low (R-squared = 0.45 and RMSE = 1.26 g/L). As opposed to the glucose and disaccharide, the arabinose content was accurately predicted (R-squared = 0.82 and RMSE = 1.77 g/L). The xylose (R-squared = 0.38 and RMSE = 2.53 g/L) and fructose (R-squared = 0.48 and RMSE = 3.37 g/L) contents were also poorly estimated. The NIR-based learning algorithms best predicted the total sugar with R-squared = 0.33 and RMSE = 26.40 g/L. Likewise, the models could not accurately predict the lactic acid (R-squared = 0.35 and RMSE = 17.95 g/L) and acetic acid contents (R-squared = 0.54 and RMSE = 1.50 g/L).

## 4 Conclusion

The spectral data were taken in NIR and MIR ranges with the aim of process monitoring of lactic acid fermentation. MIR spectra showed a consistent and explainable pattern during the fermentation of both glucose and biowaste hydrolysates. Specific peaks characteristic of acids and sugars provided reliable information on their variations. However, the NIR spectra were mixed, particularly when the biowaste hydrolysates were used as substrate. Consequently, NIR-based prediction models failed to estimate the biochemical reactions. As opposed to NIR, MIR-based predictions models showed high accuracies in the estimation of biochemical reactions for both glucose and biowaste hydrolysate media. The findings of this study indicated the potential and reliability of MIR to be further developed for inline process monitoring of lactic acid fermentation.

## Data availability

Data will be made available on request.

## CRediT authorship contribution statement

**Arman Arefi:** Writing – original draft, Methodology. **Barbara Sturm:** Writing – review & editing, Formal analysis, Conceptualization. **Majharulislam Babor:** Writing – original draft, Software, Formal analysis. **Michael Horf:** Writing – original draft, Methodology. **Thomas Hoffmann:** Writing – review & editing, Formal analysis. **Marina Höhne:** Writing – review & editing, Formal analysis. **Kathleen Friedrich:** Methodology. **Linda Schroedter:** Writing – review & editing, Formal analysis. **Joachim Venus:** Writing – review & editing. **Agata Olszewska-Widdrat:** Writing – original draft, Methodology, Formal analysis.

## Declaration of competing interest

The authors declare that they have no known competing financial interests or personal relationships that could have appeared to influence the work reported in this paper.
